# Assessing the Malaria Burden and Community Response to the Malaria Control and Management Programs in Omoro District, Northern Uganda

**DOI:** 10.1155/2024/8009447

**Published:** 2024-10-28

**Authors:** Divas Soyekwo, Elizabeth A. Opiyo, Reiginald Austin, Stephen Ochaya

**Affiliations:** ^1^Department of Biology, Faculty of Science, Gulu University 166, Gulu, Uganda; ^2^Department of Microbiology and Immunology, Faculty of Medicine, Gulu University 166, Gulu, Uganda; ^3^Department of Clinical Pathology, Uppsala Academic Hospital, Uppsala, Sweden

**Keywords:** community response, insecticide residual spraying, local herbs, malaria burden, mosquito net use, number of malaria attacks

## Abstract

**Background:** Malaria remains the leading cause of hospitalization and death in the healthcare system. This study explored the malaria burden and community response to government malaria control programs in Omoro district.

**Method:** This retrospective study involved 576 patient results from purposely selected health facilities data from health center III (HCIII) of Odek, Bobi, and Lapainat and health center IV (HCIV) of Lalogi. And prospective random section study involved 288 participants from Lutori and Lagude cells and Atyang A and Lagwaya villages who consented to answer the pretested questionnaire.

**Results:** The prevalence of malaria in Omoro district in 2018 and 2019 was 81.6% and 97.2% for hospital record positivity and community surveys, respectively. The participants had 100% knowledge of malaria signs, symptoms, and cause. The average number of malaria attacks an individual received in the district from the health facility and community data was three. Nonadherence to government control programs was associated with an increased incidence of malaria infections. From questionnaire, the proportion of people that used local herbal remedies for treatment and prevention of malaria were 21.2% of the sampled 288 participants.

**Conclusion:** The high rate of malaria attacks indicates that the area has a high prevalence of malaria-carrying mosquitoes. The increase in the proportion of malaria attacks in 2019 suggests that the burden of malaria increased compared with that in the previous year, with approximately 21% of local herbal remedies for malaria treatment and management. The findings of this study suggest that malaria attacks are associated with household size, age, sex, occupation, and the household head. Participants who did not respond positively to government programs experienced more malaria attacks. These findings can be used to develop interventions to reduce the incidence of malaria in this population.

## 1. Introduction

According to the 2022 World Malaria Report, an estimated 227 million cases of malaria have occurred worldwide in 2021 in 84 endemic countries. Twenty countries contributed up to 96% of the malaria cases. Of these, Nigeria contributed 27%, the Democratic Republic of Congo 12%, Uganda 5%, Mozambique 4%, and Niger 3%, contributing up to 51% of all global cases. Indeed, the burden of malaria is heaviest in the African region, where an estimated 90% of all malaria deaths occur [1, 2]. However, malaria deaths have decreased by 58% from 6200 to 2600 between 2000 and 2021. The total percentage of malaria-related deaths among children under the age of 5 years in 2019 was 67%.

Roll back malaria strategies play a major role in reducing malaria-related morbidity and mortality [1]. Local remedies are used to control malaria in areas such as Uganda and Nigeria [3, 4].

The National Malaria Control Program in Uganda is charged with providing quality-assured services for malaria prevention and treatment for all people in the country. This program guides malaria control efforts as outlined in the Uganda Malaria Reduction Strategic Plan (UMRSP) 2014–2020. The government of Uganda has implemented several malaria control programs, such as indoor residual spraying (IRS), use of insecticide-treated nets, test and treatment strategies, intermittent prophylactic therapy (IPT) for pregnant women, introduction of village health teams (VHTs) to manage fevers, and radio and television (TV) education programs on malaria prevention and control. In 2009, the Malaria Indicator Survey (MIS) reported a high prevalence of malaria parasites in children aged < 5 years, ranging from 5% in Kampala to 63% in the midnorthern region, with a national average of 45% [4].

Acholi subregion in northern Uganda reported 668,787 (5.16%) of total country's malaria-positive cases, while Omoro district had 83,598 (0.62%) confirmed malaria cases [5]. There is no recorded information indicating the number of malaria attacks an individual receives in a year or what local remedies are used for treatment/management. In addition, since the success of any control program and status of the diseases depends on the target community, it was also important to assess the community's response to government control programs using pretested questionnaire, the reason for this research. This study sought to establish the number of malaria attacks an individual receives in 1 year in Omoro district using the 2018 and 2019 HMIS and laboratory records, determine the local herbal remedies used by the community for the treatment and management of malaria, and assess the community's response to government malaria control programs implemented in Omoro district, northern Uganda.

## 2. Methods and Materials

### 2.1. Study Site

**Figure 1:**
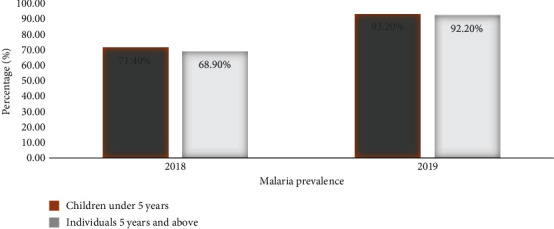
Malaria prevalence 2018 and 2019.

This study was conducted in Omoro district, in northern Uganda. The district is approximately 1200 masl and receives approximately 1300 mm of annual rainfall [5]. Omoro district is located at 2.7152° N and 32.492° E, covering a total area of 1556 km [6]. The projected population of the district by December 2020 was 201,266, with a population density of 126.2/km^2^ (District Planning Office, Omoro). The largest population comprises peasant farmers, with a small percentage of civil servants and business communities. The vegetation is savanna grassland in the entire region (Figure 1).

**Figure 2:**
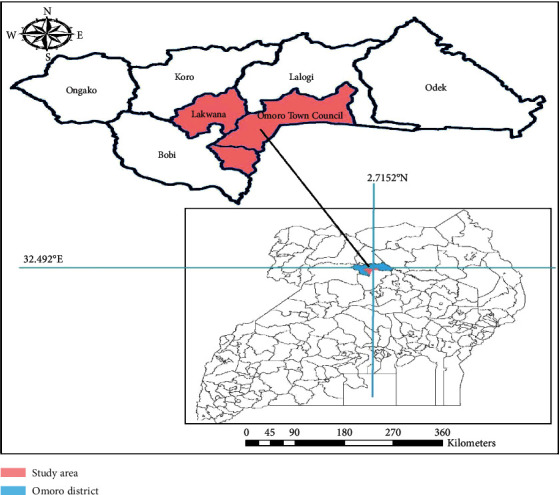
Map showing the study area within Omoro district, Uganda.

The study was conducted in Omoro town council and Lakwana subcounty, located within Omoro district, Uganda (Figure 2). The highlighted area (in pink) represents the specific study site, while the blue outline indicates the boundaries of Omoro district. Coordinates of the study area are approximately 2.7152° N latitude and 32.492° E longitude (Source: Soyekwo Divas, 2021).

### 2.2. Map Showing Study Area

The district comprises of Tochi River, with several swamps and several central forest reserves, including Opok central forest reserve, Opit central forest reserve, Lalogi central forest reserve, and Opaka central forest reserve. All these features are a great contributor to mosquito breeding sites, making Omoro district one of the districts with high malaria prevalence in northern Uganda. The most common malarial vector is *Anopheles funestus*. The district has 13 health center II, eight health center III, and one health center IV. According to the Uganda malaria annual report [6], the total number of confirmed malaria cases per 1000 population in Omoro district was the second highest with 250/1000, while other neighboring districts had less like Pader 235/1000, Oyam 188/1000, Nwoya 231/1000, Lira 70, and Gulu 281/1000.

### 2.3. Study Design

The study was retrospective, assessing malaria burden in Omoro district for the period of first January to 31th December 2018 and the first January to the 31th December 2019. The number of malaria attacks an individual receives in a year and the role of the community in the control and management of malaria were investigated. A prospective study design was used to collect quantitative community data using a questionnaire. A multistage sampling technique with simple random sampling was employed. From the list of two counties, simple random sampling was performed to obtain Omoro county. Finally, a list of 74 and 86 Lutori and Lagude households, respectively, and 77 and 68 in Atyang A and Lagwaya, respectively, was randomly sampled to obtain 24 households for administering the questionnaires.

### 2.4. Sample Size Determination

The sample size of 288 was obtained using the formula described [6].

 $$\begin{array}{c} N=\frac{Z^{2}pq}{L^{2}}=288\text{ people were considered for each objective} \end{array}$$

The total sample size for the study was 864, of which 576 were for the retrospective study of health facility records captured from laboratory record books of 2018 and 2019 and 288 were from the perspective study design of household participants of questionnaires.

### 2.5. Sampling Criteria

For the retrospective study, establishing the number of malaria attacks an individual receives in a year from 2018 to 2019 in Omoro district, the general laboratory record was used to select every third positive result. These were followed up until the end of the year, resulting in 72 confirmed malaria patients per health facility.

For the prospective study, quantitative data were collected by administering 288 questionnaires to different study participants from the sampled village households after listing them from the county to village level and then to the household level.

### 2.6. Inclusion Criteria

The hospital and district data captured only confirmed malaria cases, both by microscopy and malaria rapid diagnostic tests, irrespective of complicated and uncomplicated malaria.

Community members who provided consent to participate in the study were included as study participants to answer the questionnaires.

### 2.7. Exclusion Criteria

Omoro district health information system records with results other than malaria-confirmed tests by mRDT and microscopy were excluded. Community members who did not consent to participate in the study, households that did not have a mixture of children under 5 years, and any two other persons from 5 years and above were also excluded.

### 2.8. Study Tools

A precoded block questionnaire and informed consent form with a sign/thumbprint was developed by the researcher and translated into the local language (Luo-Acholi) for easier interpretation. The questionnaire was pretested for validity and reliability on 20 participants, six under 5 years, six 5–17 years, and eight adults, from Koch Cell, Pece Acoyo, and Laroo division in Gulu city, an area outside the study area, for its applicability before it was used to collect the actual data. Questionnaires were used to determine people's views and use of local remedies for the treatment of malaria. Prevalence records were captured from the health information system, from the statistician in Omoro district from 2018 to 2019. The number of malaria attacks was obtained by following up confirmed malaria cases from 2018 to 2019 from laboratory records using a developed data-collection tool.

### 2.9. Data Collection

Retrospective data were purposively collected from HCIII of Odek, Lapainat, and Bobi and HCIV of Lalogi based on their proximity to forests, swamps, rivers, urban areas, and grasslands to determine the number of malaria attacks in a year. Quantitative data from laboratory general record books were used by systematically selecting every third malaria positive, both complicated and uncomplicated, confirmed by both microscopy and rapid diagnostic tests of at least 24 children under 5 years and 48 for those 5 years and above from every health facility. This study was conducted for the period from January–December 2018 to January–December 2019 to avoid bias. Data on total malaria cases were obtained from the district health officer (DHO) Omoro district and statisticians.

Local herbal remedies in malaria treatment/management and community responses to government control programs were assessed using questionnaires for study participants who consented and met the inclusion criteria.

Household quantitative data from household heads (head of household: this means male (husband), female (wife), or child headed either male or female who takes responsibility for people who reside in the same compound where there are many housing units) were obtained from four villages of Lagude, Lutori in Lagude ward, Omoro town council, Atyang A, Lagwaya in Lujorongole parish, and Lakwana subcounty, all of which are in Omoro County. These participants were independent of health facility data.

### 2.10. Data Management and Analysis

To analyze both malaria prevalence and local remedies used in malaria treatment, the data obtained were summarized in an Excel sheet, coded, exported, and analyzed using the chi-square test in SPSS version 26.

To assess the community's response to government malaria control and management programs and the number of malaria attacks, quantitative data were entered into Excel sheets, cleaned, coded, analyzed using SPSS version 26 software, and analyzed with chi-square. Bivariate and multivariate analyses were performed.

### 2.11. Ethical Clearance

Ethical clearance was obtained from the Gulu University Research Ethics Committee (GUREC), and approval was obtained with the reference number GUREC 2021-105. Permission to carry out the study was obtained from the DHO, chief administrative officer (CAO), resident district commissioner (RDC) of Omoro district, facility in charges, local council ones (LC1s), and VHTs. Informed consent to administer the questionnaires was obtained from the adults and guardians of children under 18 years of age. To ensure confidentiality, the study numbers kept under restricted access and no names were used for data collection. The information obtained from the respondents was confidential.

## 3. Results

### 3.1. Sociodemographic Characteristics

The average household sizes in the four villages of Atyang A and Lagwaya in Lujorongole parish and Lakwana subcounty and villages of Lagude subward and Lutori subwards of Lagude ward, Omoro town council, were estimated at 5.7 rounded to 6. For the participants, most of the households were male headed, age brackets of 15 years were highest with low education status at the primary level, and most participants were peasants (Table 1).

For the household, in every household, three participants were purposely selected to answer questionnaire after consenting to participate. One child was under 5 years, irrespective of sex; one was above 5 to 17 years; and at least one was an adult. However, in families with a child between 5 and 17 years of age, two adults who happened to be household head were considered.

As shown in Table 2, the low possession of essential items for malaria control likely contributed significantly to several malaria attacks in Omoro district community.

Table 3 shows the associated factors for the number of malaria episodes per year (Pearson's chi-square tests). Household size, household head, age, and occupation were found to have statistically significant associations with malaria episodes per year, with *p* values below the threshold of 0.05. Factors like sex, education level, and type of housing showed no significant relationship (*p* > 0.05).

### 3.2. Malaria Prevalence

Malaria prevalence was calculated by dividing the number of malaria-confirmed positive tests by the total numbers of tests performed throughout the year, multiplied by 100. In 2018, the total number of malaria-confirmed positive tests by microscopy and mRDTs was 31,554, and out of the total tests performed of 45,429, the prevalence was 69.5%. In 2019, 127,185 tests were performed, of which 119,196 were positive, with a prevalence of 93.7%.

### 3.3. Number of Malaria Attacks an Individual Receives in Omoro District From Health Center Data

**Figure 3:**
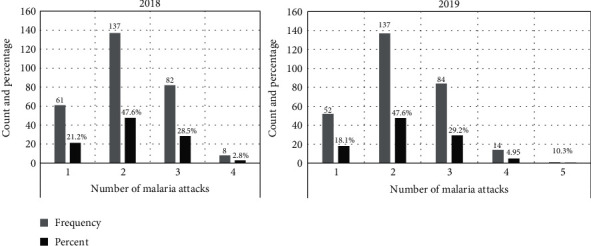
Number of malaria attacks in 2018 and 2019.

Retrospective quantitative data on the number of confirmed malaria attacks by microscopy and mRDTs an individual receives in a year using 2018 and 2019 data from laboratory record books and HMIS forms from the health center III of Lapainat, Bobi, and Odek and health center IV of Lalogi were presented. In 2018, a significant number of malaria attacks were reported; 137 (47.6%) participants had two malarial attacks per year, followed by 82 (28.5%) with three attacks per year (Figure 3). In 2019, there were 137 (47.6%) malaria attacks, followed by 84 (29.2%) attacks (Figure 3). The average number of malaria attacks experienced by the individuals in 2019 was two.

### 3.4. Number of Malaria Attacks From Households Using Research Questionnaires

**Figure 4:**
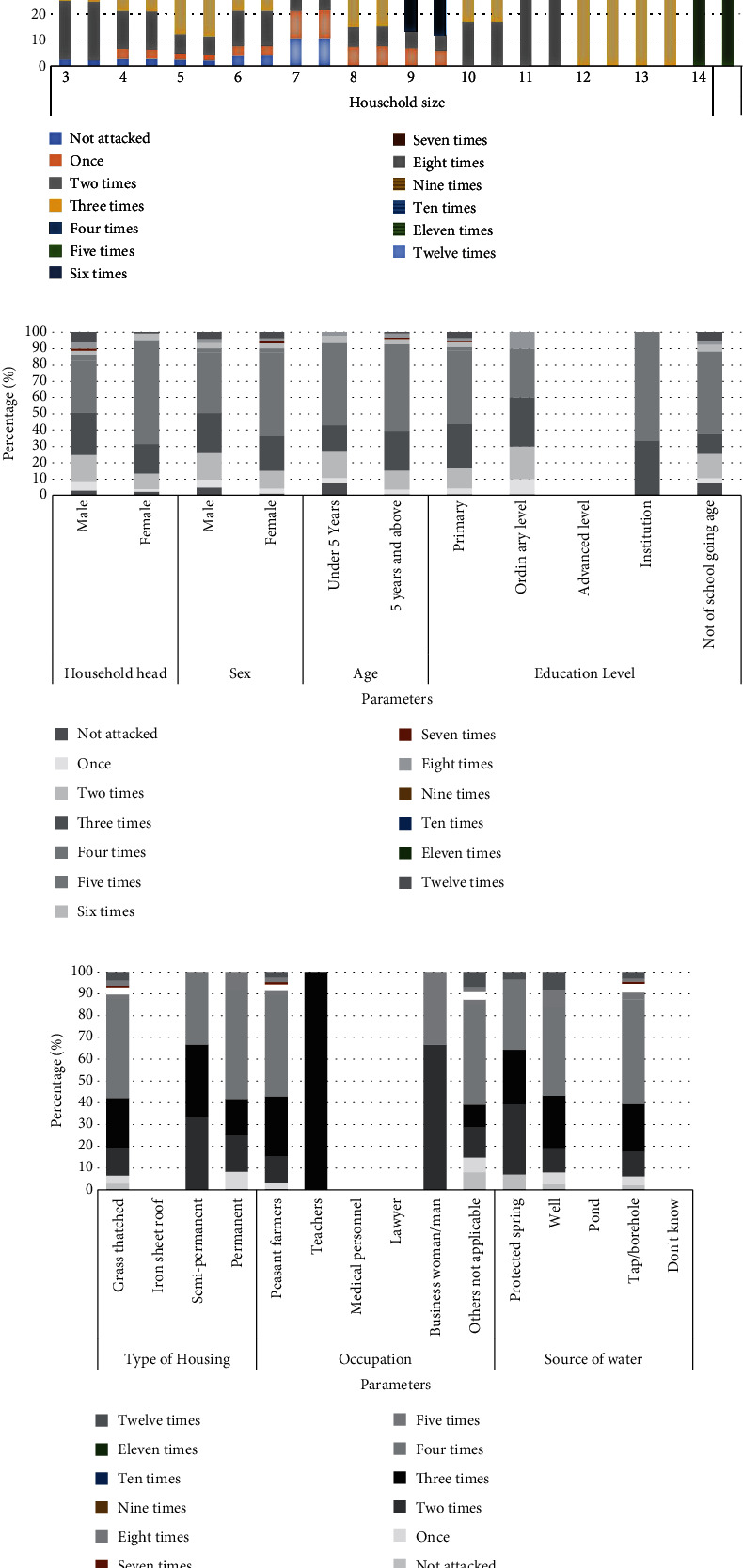
Number of malaria attacks from household questionnaires. (a) Household size. (b) Household head, sex, age, and education level. (c) Type of housing, occupation, and water source.

Questionnaires were administered to 72 households in different villages in order to assess the number of malaria attacks in the community. Data from households from April 2021 to March 2022 using research questionnaires had different values for the number of malaria attacks an individual receives in 1 year, three on average. The participants had 100% knowledge of malaria signs, symptoms, and causes. Note that the study participants were different from those examined in the laboratory records because the sampled health facilities were far from the sampled household communities. As shown in Figure 4(a), a household size of four reported four attacks in a year, and the highest number of malaria attacks was 31 (10.8%). The majority, 80 (27.8%) female-headed households, reported four episodes, and 51 (17.7%) male-headed households also confirmed the same (Figure 4(b)). Among the males, 46 (16%), and among the females, 85 (29.5%), all were attacked four times (Figure 4(b)). The majority 126 (14.7%) of those aged 5 years and above were attacked four times, followed by 60 (7.0%) three times, and 44 (7.1%) of those under 5 years were also attacked four times, as shown in (Figure 4(b)). Under the education level, the majority 82 (28.5%) from the primary level were attacked four times, followed by 49 (17%) three times, and 44 (15.3%) not of school-going age were attacked four times (Figure 4(b)).

Regarding housing type, 124 (43.1%) grass-thatched housing types were attacked four times, 62 (21.5%) were attacked three times, and 35 (12.2%) were attacked twice. In terms of occupation, 91 (31.6%) peasant farmers were attacked four times, followed by 54 (18.8%) three times, and 40 (13.9%) children aged < 14 years were attacked four times (Figure 4(c)).

Pearson chi-square test was performed to assess the significant association between household size and the number of malaria attacks. There was a significant association between malaria attacks and household size (*χ*^2^ = 151.624, *p* < 0.001), head of household (*χ*^2^ = 37.643, *p* < 0.000), sex (*χ*^2^ = 10.00, *p* < 0.001), age (*χ*^2^ = 96.590, *p* < 0.000), and occupation (*χ*^2^ = 55.168, *p* < 0.001).

### 3.5. Local Herbal Remedies Used for Malaria Treatment/Management

The use of local herbal remedies was assessed using questionnaires completed by 288 participants who consented to participate in the study according to the sample size. According to our findings, 61 patients (21.2%) used local herbal remedies for malaria treatment and management. However, those who were able to use it were less attacked by malaria than their counterparts who did not. Local herbal remedies were categorized into three categories: preventive/prophylaxis such as *Cymbopogon citratus* and *Carica papaya*, Eucalyptus shoots, and *Mangifera indica* roots and shoots. These were prepared by pounding, and the ingredients were mixed with water and administered orally. Adults are recommended to take one cup of half a liter, whereas children are recommended to take 5 teaspoons twice a month. *Vernonia amygdalina* was prepared by pounding, mixed with water, and taken twice a month, adults' one cup of half liter, whereas children take 5 teaspoons to half liter depending on age. Treatment/management include *Cucurbita maxima*-acula, *Aloe vera*, *Bidens pilosa*, *V. amygdalina*-labwori, *Rhus natalensis-*aywaca, Neem, and *Sarcocephalus latifolius*-oculub. These were prepared by pounding, mixing with clean water, and taking twice a day for 3 days. Adults took half a liter of one full cup, whereas children took 5 teaspoons to half a cup twice a day for 3 days.

To examine the significance of the local herbs used and malaria attacks, a chi-square test was conducted. The chi-square value obtained was 86.947 at the 5% level of significance and 90 degrees of freedom. It denotes there was no significant association between local herbs used and prevalence of malaria (*p* value = 0.572).

### 3.6. Community Responses to Government Malaria Control Programs

The government of Uganda has been implementing malaria control programs, such as IRS, use of insecticide-treated mosquito nets, IPT for pregnant mothers, and test and treatment strategies. A questionnaire was administered to 288 participants to assess their community's responses. Having bicycles for quick response in case one falls sick of malaria and having radio and TV by listening to and watching government education programs on malaria control were some of the assessment tools used to evaluate the community response to government programs. As shown in Table 4, those who did not test for malaria and did not replace their mosquito nets were ranked highest among those who did not respond positively to government programs. Logistic regression tests were performed to assess association between malaria infection and adherence to government control programs. There was a significant association between the condition of the mosquito nets and the number of malaria attacks (*p* = 0.046) (Table 4).

There was a significant association between the condition of the mosquito net and malaria attacks at 0.46 and chi-square value of 0.04 of those who owned a television.

Community response was associated with willingness to take up the IRS. Respondents who knew the reason for spraying (to kill mosquitoes) and the frequency of spraying (after every 6 months) were considered knowledgeable (Table 5).

Those who use water from protected spring were 0.36 times less likely to have mosquito nets at 95% CI (0.55–0.32) and chi-square of 0.00 showing significance. Those who had radios were 4.6 times more likely to have mosquito nets compared to those who did not have a 95% CI (1.18–15.47) sig. 0.027, which was statistically significant (Table 6).

## 4. Discussion

This retrospective and prospective study investigated the malaria burden and community response to government malaria control programs that could be used to create or improve malaria prevention and control programs. The data were collected from the HCIII of Odek, Bobi, and Lapainat and HCIV of Lalogi, at the district headquarters. From this research, much as government interventions have been put in place to control malaria, coverage remains low. A lower malaria prevalence was observed in 2018 than in 2019. This could be attributed to the increasing number of mosquito breeding sites with stagnant water and bushes, which agrees with a previous report [7, 8].

Regarding sociodemographic characteristics, using the chi-square test, there was a significant association between household size and the number of malaria attacks an individual receives in a year. In this study, a household size of four had the highest number of malaria attacks, followed by a household size of six. This contradicts the results of the previous studies [5, 9], where the higher the household size, the higher the risk of malaria infection because mosquitoes are attracted by high carbon dioxide concentrations, as previously noted. The community's behavior of not using mosquito nets and reluctance to purchase would also have contributed to the high prevalence of malaria. The literacy levels of participants influenced malaria prevalence and the number of malaria attacks in a year because the less educated had a low understanding of the causes and preventive measures of malaria compared to those who had studied beyond the primary level. This finding is consistent with the previous reports [10, 11]. On a macroscale, malaria is considered to be a disease of the poor. However, when it comes to the microscale, it becomes difficult to analyze because of less inconsistent evidence [11]. The distribution of occupation percentages revealed that most participants were unable to seek malaria treatment from other private health facilities. Further assessing the wealth level, 195 had no radios in their homes to listen to education programs for malaria prevention and control, hence the high prevalence. A similar observation has been reported previously [12, 13].

The high prevalence rates of malaria in the Omoro district community were associated with age, sex, source of water, occupation, household head, and radio possession in 2018 and 2019, respectively. The year 2019 had the highest prevalence of malaria in Omoro district and in the northern and midnorthern regions of Karamoja [14]. According to sources from Omoro district, the government had to employ other medical staff such as medical officers, clinicians, and nurses to support almost all health facilities in the district [15]. In each household, at least one or two members had suffered from malaria once or more at the beginning of the year.

Community questionnaires revealed up to 12 malaria attacks. Probably, the main cause could be the alternative treatment options of using local herbs for malaria treatment without confirmation by microscopy after a previous malaria attack, which agrees with a previous study [16].

The mean value of malaria attacks an individual receives in a year in 2018 and 2019 was two, and the mean value of malaria attacks an individual receives in a community in a year was three. This study did not establish the number of deaths during the study period in 2018 and 2019, which might not have provided the accurate number of malaria attacks that an individual receives in a year. It was also evident that Omoro district community sought treatment from nearby government health facilities with few visits to clinics or other hospitals, such as St. Mary's Hospital Lacor, of which there was no overlap of data collected for the number of malaria attacks an individual receives in a year. This study agrees with the study [5], which revealed that hospital data are not very accurate because of poor documentation and alternative seeking treatment options for other patients.

Regarding the local remedies used by the community for malaria treatment and management in Omoro district, household data showed that a small percentage of individuals use local remedies for malaria treatment and management. Most people lacked knowledge of the dose, concoctions, and relevance of local herbal remedies. Among the local remedies used was *B. pilosa* leaf extracts used to cure malaria, which conforms to the previous studies [17, 18]. The use of *A. vera* and *C. papaya* conformed to a previous study [18]. The use of *M. indica* roots and bark, *C. citratus* leaves, *V. amygdalina*, *A. vera*, and *C. papaya* leaves agrees with the previous research [19].

In this study, the participants had knowledge of malaria because they correctly associated mosquitoes with malaria transmission. There are still misconceptions that malaria is caused by drinking dirty water, which coincides with the previous findings [20]. On another note, according to the research findings in this study, the communities' sociocultural beliefs contradict [21] that women could confuse signs of pregnancy as malaria. In this study, they were able to differentiate between malaria and pregnancy.

Furthermore, in this study, the knowledge of malaria control and prevention was attributed to possession of radio or television. Studies have shown that improved community knowledge of malaria and its source of transmission promotes preventive and personal protection practices among the affected community, as documented elsewhere [1, 16]. This provides an opportunity for any malaria prevention and control intervention to be utilized for maximum effectiveness.

Regarding the community's response to government malaria control programs, Omoro district was affected by several factors. In this study, the number of malaria attacks an individual receives per year was related to the household head, household, size, age, sex, education level, source of water, and occupation. However, these categories regularly used insecticide-treated mosquito nets to prevent malaria attacks, whereas others were reluctant to use them effectively. There was sole dependence on the government to replace worn-out/torn mosquito nets, of which, at some point, when there was a delay, people stayed without mosquito nets, resulting in a high resurgence of malaria in 2019. Generally, the entire community was compliant with the government malaria control of test and treatment, prescribed complete malaria doses, mosquito net use, and use of IRS. With low possession of radios, bicycles, television, and peasant farmers living in grass-thatched houses, most people sought treatment from government health facilities with few visits to clinics or other hospitals such as St. Mary's Hospital Lacor, of which malaria drugs sometimes run out of stock, leading to malaria resurgence. IRS was performed in 2009 in most households, when northern Uganda had the highest malaria prevalence in the country. After the IRS exercise, IPT for pregnant women intensified, resulting in a low prevalence of malaria in the region in 2016. There was a resurgence of malaria after the withdrawal of the IRS, particularly in 2015.

## 5. Conclusion

This study found that the average number of malaria attacks an individual receives in Omoro district is three. This was attributed to the poor adherence of the community to government malaria control programs of improper use of ITNs and LLINs, waiting only for the government to supply ITNs. The number of malaria attacks from household quantitative data indicated that others were being attacked by malaria up to 12 times a year, confirmed using mRDTs, and by microscopy, the maximum was five times a year. From the questionnaires, most participants reported that Artemisinin-based Combination Therapy (ACTs) was the treatment of choice given to them. Others used local herbs. The herbs used were *S. latifolius*-oculub, *R. natalensis-*awawaca, *B. pilosa*, and *V. amygdalina*-labwori. These were used in the early stages of noticing signs of fever, headache, and vomiting before seeking medical attention.

## Figures and Tables

**Table 1:** Demographic characteristics of research participants of questionnaires.

**Variable**	**Characteristics**	**Frequency**	**Percentage**
Household head	Male	161	55.9%
	Female	127	44.1%

Age	<1 year	31	10.8%
	1–4 years	67	23.3%
	4–14 years	78	21.7%
	> 15 years	112	38.9%

Education level	Not school going	94	32.6%
	Primary	181	62.8%
	Ordinary level	19	3.5%
	Advanced level	0	0.0%
	Institution	3	1%

Occupation	Peasant farmers	196	68.1%
	Teachers	2	0.7%
	Businessmen/women	3	1%
	Children < 14 years	87	30.2%

**Table 2:** Essential items of response to government malaria control programs.

**Item**	**Possession**	
	**Yes**	**No**
Radio	93 (32.3%)	195 (67.7%)
Bicycle	89 (30.9%)	199 (69.1%)
Television	12 (4.2%)	276 (95.8%)
Mosquito net use	136 (47.2%	152 (52.8%)

**Table 3:** Associated factors for number of malaria episodes per year.

**Pearson chi-square tests**		
Household size	Chi-square	151.624
	Sig.	0.001⁣^∗^
Household head	Chi-square	37.643
	Sig.	0.000⁣^∗^
Sex	Chi-square	10.638
	Sig.	0.301
Age	Chi-square	96.590
	Sig.	0.000⁣^∗^
Education level	Chi-square	27.191
	Sig.	0.454
Type of housing	Chi-square	6.158
	Sig.	0.996
Occupation	Chi-square	55.168
	Sig.	0.001⁣^∗^

*Note:* This table shows significant association between household size, head, age, and occupation.

⁣^∗^The chi-square statistic is significant at the 0.05 level.

**Table 4:** Summary of bivariate tests for community responses to government control programs.

**Response to government control programs**	**Yes**	**No**	**Odds ratio**	**95% CI**	**Chi-square**
	**Count/%**	**Count/%**			
Use mosquito nets	136/47.2	152/52.8	0.871	0.83–0.91	0.08
Condition of mosquito net	148/51.4	140/38.6	1.382	1.30–1.46	0.16
Replacement of mosquito net	58/20.1	230/79.9	1.892	1.86–1.96	0.10
Was insecticide residual spraying (IRS) done?	96/33.3	192/66.7	0.906	0.87–0.94	0.070
IPT during antenatal visit	128/44.4	160/55.6	1.813	1.77–1.86	0.09
Do you own a radio?	93/23.3	195/67.7	0.323	0.27–0.38	0.11
Do you own a bicycle?	89/30.9	199/69.1	0.309	0.26–0.36	0.10
Do you own a television?	12/4.2	276/95.8	0.042	0.02–0.06	0.04

**Table 5:** Comparison of respondents to IRS response with tools to respond to malaria control.

**Respondents**	**Odds ratio**	**95% confidence interval**	**Chi-square**
Attended formal education versus no formal education	3.6	1.81–7.04	5.26
Twenty-two years above versus below 22 years of age	4.6	2.99–7.24	4.25
Possession of radio, TV, and bicycle and those without	0.6	0.34–0.94	0.6
Iron sheet houses and grass thatched	1.5	1.04–2.24	1.2
IRS not done in their houses	0.2	0.09–0.27	0.18
No knowledge of IRS	0.4	0.26–0.76	0.5
Knowledgeable about IRS	0.4	0.27–0.65	0.38

**Table 6:** Multivariate analysis of community response to government control programs.

**Response of do you have a mosquito net**	**Odds ratio**	**95% CI**	**Chi-square**
Sex			
Male	0.55	0.24–1.29	0.17
Age			
5–14 years	0.40	0.14–10.9	0.58
< 1 year	1.38	0.26–7.16	0.70
> 15 years	0.28	0.10–7.74	
Education			
Institution	1		0.68
Not school going	1.65	1–7.12	
Primary	1		
Ordinary level	1		
Housing type			
Permanent	1		
Semipermanent	1		
Use mosquito nets	0.871	0.83–0.91	14.350
Occupation			
Business	1		
Peasant farmers	0.58	0.16–2.13	0.41
Teachers	1		
< 14 years	1		
Condition of mosquito net	1.382	1.30–1.46	28.400
Source of water			
Protected spring	1	1.86–1.96	12.136
Well	0.13	0.55–0.32	0.000
Tap/borehole	1		
Was IRS done? Yes	1.66	0.87–0.94	11.740
IPT during antenatal visit—yes	1.59	0.39–6.52	13.203
Do you own a radio? Yes	4.27	1.18–15.47	0.027
Do you own a bicycle?	0.309	0.26–0.36	15.351
Do you own a TV? Yes	3.69	0.24–56.91	0.350

## Data Availability

The authors have nothing to report.
